# Treatment planning of epithelial ovarian cancers using helical tomotherapy

**DOI:** 10.1120/jacmp.v10i4.3003

**Published:** 2009-10-07

**Authors:** Swamidas V Jamema, Umesh Mahantshetty, Vineeta Goel, Reena Engineer, Deepak D Deshpande, Rajiv Sarin, Shyam Kishore Shrivastava

**Affiliations:** ^1^ Department of Medical Physics Tata Memorial Hospital Tata Memorial Centre Mumbai India; ^2^ Radiation Oncology Tata Memorial Hospital Tata Memorial Centre Mumbai India; ^3^ Department of Radiation Oncology Advanced Center for Training Research and Education in Cancer Tata Memorial Center Kharghar, Navi Mumbai India

**Keywords:** whole abdomen radiotherapy, helical tomotherapy, ovarian cancer

## Abstract

Whole abdomen radiotherapy (WAR) for epithelial ovarian cancer, though effective, has been used sparingly due to inadequate target coverage and poor sparing of organs at risk (OAR) leading to significantly higher toxicities. Newer radiation techniques have shown potential for significant improvement in the therapeutic ratio. The purpose of this study was to evaluate helical tomotherapy (HT) for WAR. The objective parameters were to obtain uniform and adequate target coverage with maximum OAR sparing. HT plans were generated for five patients with field width of 5.0/2.5 cm, modulation factor of 3.5/3.0, and a pitch of 0.3. A dose of 25 Gy in 25 fractions was prescribed to the abdomen with a simultaneous boost of 45 Gy in 25 fractions to the pelvis. Dose‐volume parameters and various indices were analyzed and compared. Mean volume (standard deviation) of abdominal and pelvic PTV (planning target volume) was ${6630\;\pm \;450\;\text{cm}}^{3}$ and ${1235\;\pm \;98\;\text{cm}}^{3}$, respectively. Mean length of PTV in cranio‐caudal direction was 41 ± 4 cm. Volume receiving 95% and 107% of the prescription dose (V95% and V107%) was 95.6 ± 2.7% and 2.6 ± 0.5% for abdominal‐PTV, and 95.7±2.4% and 0% for pelvic‐PTV, respectively. Homogeneity and conformity indices were 17.5±1.7,1.2±0.03 for abdominal PTV, and 5.2±0.7,1.1±0.02 for pelvic‐PTV, respectively. Median dose received by the kidneys, liver and bone marrow was 9.6±1.2Gy,17±2.7Gy and 22±1.4Gy, respectively. HT achieves an excellent coverage of WAR target with simultaneous pelvic boost and better organ (kidneys and liver) sparing. HT for WAR has the potential as consolidative therapy; this is being evaluated further in a phase II cohort study in epithelial ovarian cancers.

PACS number: 87.53 Kn, 87.55. D‐, 87.55.dk.

## I. INTRODUCTION

Epithelial ovarian cancer (EOC) is a surface malignancy with predilection for transperitoneal and transcoelomic spread. Despite multiple surgeries and chemotherapy (standard treatment), 60%‐70% abdomino‐pelvic recurrences have been reported.^(1)^ Various consolidative therapies, including systemic or intra peritoneal chemotherapy and radiation therapy, have been tried. Radiation in the form of whole abdomen radiotherapy (WAR), either as adjuvant treatment after maximal safe cytoreductive surgery or as salvage after chemotherapy failure, has been tried in the past with limited success.^(^ ^2^ ^,^ ^3^ ^)^ Though effective, WAR is used sparingly due to concerns regarding inadequate coverage of large target volume and poor sparing of organs at risk (OAR) leading to significantly higher toxicities.^(^ ^4^ ^,^ ^5^ ^)^

The target volume for WAR is not only large but also complex in shape. It includes the whole of abdomino‐pelvic cavity with all its contents within the peritoneal reflections including diaphragm, liver capsule and surface, gallbladder, spleen, capsule of kidneys, stomach, pancreas, bowel, vaginal vault, retroperitoneal and pelvic lymph nodes (LN). Traditionally, anterior/posterior beam arrangement was used with partial kidney and liver blocks, which produces a highly inhomogeneous dose distribution.^(^ ^6^ ^,^ ^7^ ^)^ To improve the dose homogeneity within the target and OAR sparing, moving strip technique was used with limited success.^(3)^

Recent advances in radiotherapy techniques allow a significant improvement in the therapeutic ratio of WAR. Linear accelerator (LA) based intensity‐modulated radiotherapy (IMRT) and volumetric intensity‐modulated arc therapy (IMAT) have shown potential to achieve uniform dose delivery to the target with significant sparing of OARs.^(^ ^8^ ^,^ ^9^ ^)^ However these techniques have inherent limitations such as multiple isocentres and intensity feathering. WAR using helical tomotherapy (HT) with homogeneous dose distribution and better sparing of OARs has been shown to have fewer limitations.^(10)^ HT has the advantages of treating longer field lengths, to a maximum of 160 cm. It uses a number of beam projections with high modulation which can provide uniform dose distribution to the target with sparing of OARs. HT also has image‐guidance capability using mega‐voltage CT (MVCT). This permits detection and correction of setup errors by verifying target and OAR positions, especially while treating large and complex target volumes with high dose gradients. Total body irradiation and craniospinal irradiation with HT have been implemented successfully in clinics.^(^ ^11^ ^,^ ^12^ ^)^ With a goal of achieving homogenous dose distribution to WAR target as well as organ sparing of kidneys, bone marrow and liver with HT, we undertook this dosimetric study.

## II. MATERIALS AND METHODS

Abdominopelvic computer tomography (CT) datasets of five patients of postoperative gynaecological cancers who underwent routine postoperative radiotherapy were used for this dosimetric study.

### A. Imaging

With patients positioned supine, arms above head, and immobilization with a vacuum cradle (Medical Intelligence Technologies, Canada) or thermoplastic mould and knee rest, planning CT images were acquired using a Siemens Sensation Multislice CT scanner (Siemens Medical System, Germany). After emptying the bladder, all patients were instructed to drink 500–1000 ml of water, 45 minutes prior to CT scan (for constant moderate bladder filling). CT scans were obtained with intravenous contrast from the mid‐thorax to mid‐thighs with 5 mm contiguous slice thickness and transferred via network to Coherence Dosimetrist Contouring Workstation (Siemens Medical System, Germany), where the target volumes and OARs were delineated.

### B. Volume delineation

The target volumes and OARs were delineated in accordance with International Commission of Radiation Units and measurements (ICRU) 50 and 62 recommendations.^(^ ^13^ ^,^ ^14^ ^)^ Clinical target volume (CTV) included the entire peritoneal cavity from the diaphragm to vaginal vault and was divided into abdomen and pelvis. Abdominal CTV included the entire peritoneal cavity with bowel and mesentery, liver capsule with surface of liver parenchyma, the undersurface of liver, the abdominal surface of the diaphragm, and the anterior‐lateral surfaces of both the kidneys. Pelvic CTV was contoured from L5‐S1 vertebral junction to include pelvic lymph nodal regions, pouch of Douglas and vaginal vault. Abdominal planning target volume (PTV) was drawn with differential margins to CTV: 1.5 cm cranially (for diaphragm movements) and 0.5 cm in all other directions. Similarly, pelvic PTV was drawn with 1 cm margin in the caudal direction and a 0.5 cm margin in all other directions. Various OARs contoured included kidneys, liver, bone marrow (ribs, vertebrae, pelvic bones and upper end femurs), urinary bladder, rectum, and heart. A structure named “normal liver” was created just inside liver PTV to control doses within non‐PTV liver parenchyma.

### C. Treatment planning

The CT datasets along with the contours of each patient were exported to Hi‐Art Tomotherapy (Version 2.2, TomoTherapy Inc., Madison, WI, USA) treatment planning system (TPS). An IMRT plan based on HT was generated for all five patients. A total dose of 25 Gy in 25 fractions at 1 Gy per fraction was prescribed to the abdominal PTV with simultaneous boost of 45 Gy in 25 fractions at 1.8 Gy per fraction over 5 weeks (5 fractions per week) was planned for pelvic PTV. Various dose levels for varying indications (consolidation / salvage / palliative) have been used in the past.^(^ ^15^ ^,^ ^16^ ^)^ The literature review of dose response suggests that whole abdominal radiation of more than 22.5 Gy is associated with higher small bowel complications requiring surgeries. Moreover, in our proposed phase II study, WAR with HT will be used as consolidation after surgery and chemotherapy in complete responders and those with minimal residual disease (<1cm). The current dosimetric study reported here has evaluated WAR as consolidation therapy in five patients. A dose of 45 Gy was prescribed to pelvis, as the pelvis is the major site of relapse, and higher radiation doses are well‐tolerated by the pelvis.^(^ ^10^ ^,^ ^17^ ^)^ Hence, a dose of 25 Gy to the whole abdomen and a simultaneous boost to the pelvis to a total dose of 45 Gy was planned.

HT plans were obtained using a field width of 2.5 and 5.0 cm and a modulation factor of 3.0 and 3.5, respectively. A pitch of 0.3 was used for all the plans. Principles of HT, inverse planning algorithm, and optimization parameters have been described previously in detail.^(^ ^18^ ^,^ ^19^ ^)^. The initial beamlet calculations were carried out with relaxed constraints such that a maximum number of beam projections was chosen for further optimization. Initially, PTV coverage was given a higher priority over OAR sparing during optimization. After obtaining the optimal PTV coverage, OARs in the following order – kidneys, liver and bone marrow – were penalized such that the dose was reduced to the minimum without compromising the PTV coverage, homogeneity and conformity. The acceptable criteria of the plan are as follows: a) 100% of the PTV should be covered by at least 95% of the prescription isodose; b) no volume of PTV was allowed to exceed dose more than 107% of the prescription dose; c) minimum dose achievable to the OARs while respecting a) and b). Once the acceptable plan was obtained, full scatter dose calculation was carried out. Since the volumes were almost similar, class solution with a set of constrains identified, reduces the planning time. To control the dose spillage outside the PTV, dummy organs were made with Boolean operations such as skin minus PTV, which were used during optimization. The nominal dose rate for these plans was 850cGy/min at 85 cm SSD for a field size of $5\times 30\;{\text{cm}}^{2}$.

### D. Evaluation parameters

For PTV, V95%, VI07% (volume covered by the 95% and 107% isodose lines, respectively), $D_{\max}$ (maximum dose), $D_{\min}$ (minimum dose) and $D_{\text{mean}}$ (mean dose) were compiled. $D_{\max}$ and $D_{\min}$ were defined as the volume received by more than 1% and 99% of the PTV, respectively. Conformity and homogeneity indices were also evaluated. The homogeneity index (HI) and conformity index (CI) were calculated according to the following formulae:^(20)^

(1) $$\text{HI}=(D_{5}-D_{95})/D_{\text{mean}}\times 100$$

where $D_{5}$ and $D_{95}$ are the doses at which cumulative DVH intersected with 5% and 95% of the volume, respectively.

(2) $$\text{CI}=1+(V_{95}-V_{\text{ptv}})/V_{\text{ptv}}$$

where $V_{\text{ptv}}$ refers to the volume of the PTV and $V_{95}$ refers to volume encompassed by 95% isodose surface.

For OARs, the maximum, minimum, mean, and median doses, and the $V_{65\%}$ and $V_{35\%}$, (volume receiving 65% and 35% of the prescribed doses, respectively) received by the kidney, bone marrow, liver, spinal cord, rectum, urinary bladder and heart were evaluated.

## III. RESULTS

**Figure 1:**
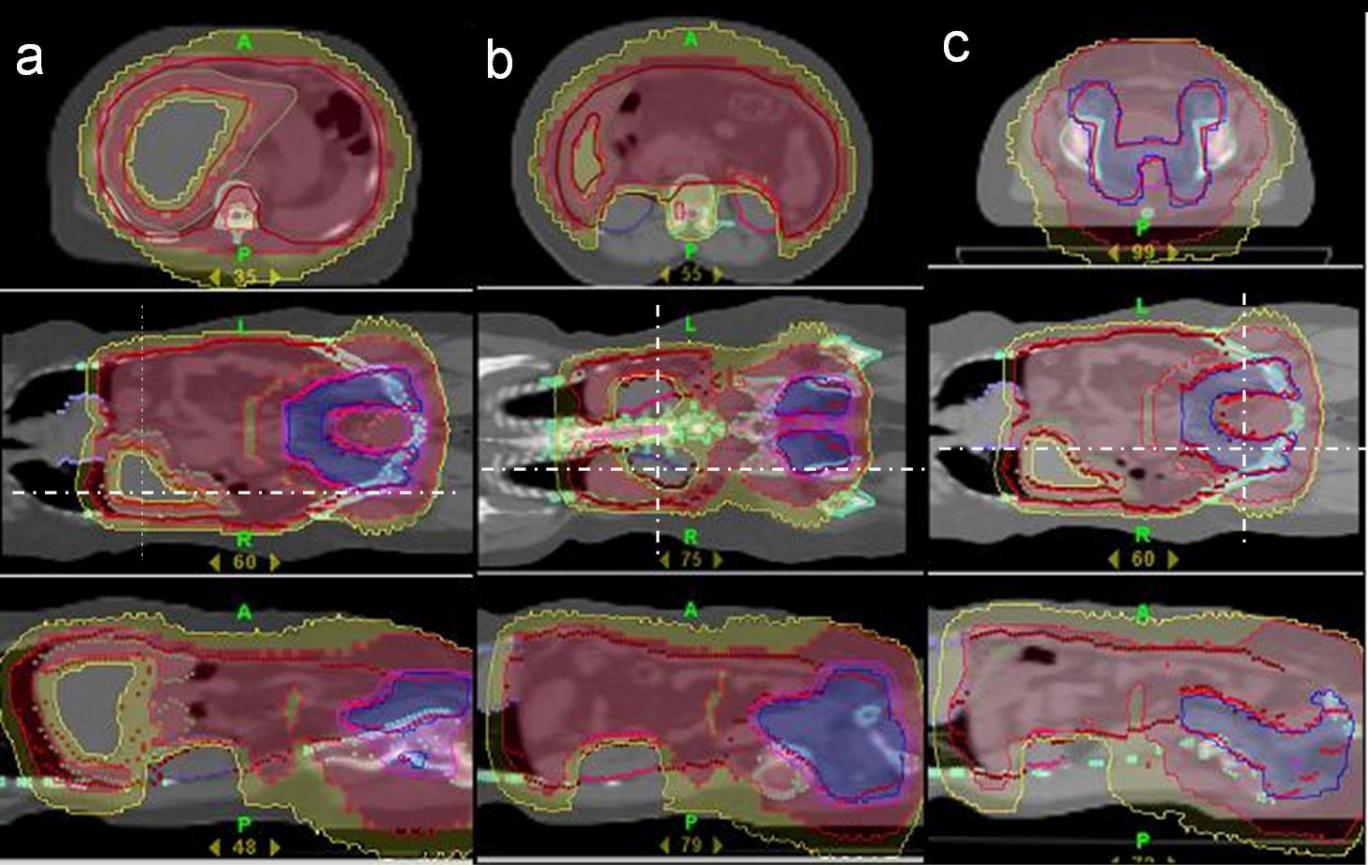
The isodose distribution at various axial sections: (a) at the level of the liver; (b) at the level of the kidneys; (c) at the level of the rectum and bladder. Red indicates 25Gy, yellow 20Gy, Blue 45Gy.

**Figure 2:**
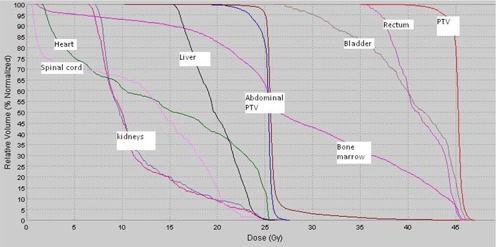
Cumulative dose volume histogram of various organs of a representative patient.

Figures 1 and 2 show a typical isodose distribution of a representative patient at the level of the liver, kidneys, rectum, bladder and the dose volume histogram. Table 1 illustrates the dose volume statistics of the PTV and the OARs. All the dose volume parameters presented for target and OARs are the mean values of five patients investigated in this study.

**Table 1:** Dose volume parameters of the PTV and the OARs.

*Parameters*			*WA‐PTV*					*Pelvic PTV*
Volume cc			6270±470					1235±98
$D_{\max}$			35.4±2.1					46.1±1.1
$D_{\min}$			24±1.6					41.1±1.5
$D_{\text{mean}}$			25.61±1.5					45.07±2.3
$V_{95\%}$			95.6±2.7					95.7±2.4
$V_{107\%}$			2.6±0.5					0
HI			17.5±1.7					5.2±0.7
CI			1.202±0.03					1.112±0.02
*OAR*	*Kidney*	*Normal Liver*	*Liver*	*Bone marrow*	*Heart*	*Spinal cord*	*Rectum*	*Bladder*
Volume cc	115±14	510±54	1017±167	1345±165	500±65	31±4	40.6±4.5	120±17
$D_{\max}\;\text{Gy}$	25.89±2.1	24.6±1.5	26.1±1.7	48±3.4	24.93±1	24.7±1.1	43±1.2	48.8±1.5
$D_{\min}\;\text{Gy}$	6.5±0.7	13.1±1.1	13.07±2.3	1.2±0.01	4.4±0.5	0.7±0.05	35±3.2	24.3±2.1
$D_{\text{mean}}\;\text{Gy}$	10.8±0.8	18.3±1.4	25.2±0.8	24.1±1.6	14±2.2	11.5±1.7	40.5±1.2	39.3±2.1
$D_{\text{median}}\;\text{Gy}$	9.6±1.2	17.0±2.7	24.09±2.6	22±1.4	13.1±1.2	12.8±1.1	40.3±1.8	39.5±2.2
$D_{65\%}\;\text{Gy}$	7.8±0.9	17.5±3.0	24±3.2	24±2.3	7±1.1	10±1.2	40±2.1	37.5±1.8
$D_{35\%}\;\text{Gy}$	10.5±1.1	20.5±2.0	24.5±2.8	33.2±4.6	21±1.7	16±0.9	42±2.7	43±2.3
$V_{10\text{Gy}\;\%}$	55±3	100±1	100±1.5	90±1.6	55±1.8	60±1.7	100±0.5	100±0.6
$V_{20\text{Gy}\;\%}$	9±0.6	35±1.3	92±2.1	78±3.2	36±1.1	9±0.9	100±0.6	100±0.7

Modulation factor=3.5(2.47),field width=5.0cm,pitch=0.3. Values presented are mean ± standard deviation.

### A. dose volume parameters of PTV

The mean (range) abdominal and pelvic PTV volumes were 6630 (6190–7135) cm^3^, and 1235 (1456–1100) cm^3^, respectively. The mean length of combined PTV in cranio‐caudal direction was 41 (46–38) cm. Evaluation of the dose volume histogram reveals excellent coverage in both abdominal and pelvic PTV. Mean (standard deviation) $V_{95\%}$ and $V_{107\%}$ was 95.6±2.7% and 2.6±0.5% for abdominal PTV, while it was 95.7±2.4% and 0% for pelvic PTV, respectively. $D_{\max},\;D_{\min}$ and $D_{\text{mean}}$ were 35.4±2.1Gy,24±1.6Gy, and 25.61±1.5Gy for abdominal PTV, while it was 46.1±1.1Gy,41.1±1.5Gy, and 45.07±2.3Gy for pelvic PTV, respectively. Similarly, HI and CI were 17.5±1.7 and 1.2±0.03, and 5.2±0.7 and 1.1±0.02 for abdominal and pelvic PTV, respectively. $V_{107\%},\;D_{\max}$ and HI were found to be on the high side for abdominal PTV due to its overlap with the pelvic PTV.

### B. Dose volume parameters of OARs

The mean volumes and various doses received are detailed in Table 1.

#### B.1 Kidneys

Mean and median doses received by the kidneys were 10.8±0.8Gy and 9.6±1.2Gy, respectively. Mean $V_{20}$ and $V_{10}$ was 9±0.6% and 55±3%, while $D_{65\%}$ and $D_{35\%}$ was 7.8±0.9Gy and 10.5±1.1Gy, respectively. The $D_{\max}$ and $D_{\min}$ received by the kidneys were 25.89±2.1Gy and 6.5±0.7Gy, respectively.

#### B.2 Liver

The median dose received by the ‘normal liver’ and whole liver was 17±2.7Gy and 24.09±2.6Gy, respectively. Mean $D_{65\%}$ received by the normal liver and the whole liver was 17.5±3.0Gy and 24±3.2Gy, while mean $D_{35\%}$ was 20.5±2.0Gy and 24.5±2.8Gy, respectively. Mean $D_{\max}$ and $D_{\min}$ dose received by the normal liver were 24.6±1.5Gy and 13.1±1.1Gy, while for the whole liver it was 26.1±1.7Gy and 13.07±2.3Gy, respectively.

#### B.3 Bone marrow

Mean $D_{\max}$ and $D_{\min}$ for bone marrow was 48±3.4Gy and 1.2±0.01Gy, respectively. $D_{\text{mean}}$ and $D_{\text{median}}$ for bone marrow were 24.1±1.6Gy and 22±1.4Gy, respectively. Mean $D_{65\%}$ and $D_{35\%}$ received by the bone marrow was 24±2.3Gy and 33.2±4.6Gy, respectively. $V_{20\text{Gy}}$ of bone marrow was observed 78 as ±3.2%, while $V_{30\text{Gy}}$ was 40±1.5% of prescription dose of pelvic PTV.

#### B.4 Heart and spinal cord

Mean $D_{\max}$ and $D_{\min}$ received by the heart was 24.93±1Gy and 4.4±0.5Gy, respectively. $D_{\text{mean}}$ and $D_{\text{median}}$ was 14±2.2Gy and 13.1±1.2Gy, respectively. Mean $D_{65\%}$ and $D_{35\%}$ were 7±1.1Gy and 21±1.7Gy, respectively. Similarly, mean $D_{\max}$ of spinal cord was 24.5±1.5Gy.

#### B.5 Rectum and bladder

Mean dose to the bladder and rectum was found to be 39.3±2.1Gy and 40.5±1.2Gy, respectively. Mean $D_{\max}$ and $D_{\min}$ of rectum and bladder were 43±1.2Gy and 24.3±2.1Gy, and 48.8±1.5Gy and 24.3±2.1Gy, respectively.

### C. DVH parameters for change in beam width and modulation factors

No difference was found in the dose volume parameters of PTV with the change in the field width from 2.5 cm and 5.0 cm. For kidneys, a reduction of 1.1 Gy in median dose was observed with the change of beam width from 5.0 cm to 2.5 cm. Mean $V_{10\text{Gy}}$ was found to be 13% less in 2.5 cm beam width plan, while $V_{20\text{Gy}}$ was similar – 9% in a 5.0 cm beam width plan. No difference in the dose volume parameters was found for liver with the change in the field width. For bone marrow, 2.5 cm field width plan offered a marginal improvement in sparing. $V_{20\text{Gy}}$ and $V_{30\text{Gy}}$ were decreased by 7% and 4% respectively in 2.5 cm field width as compared to 5.0 cm field width. For organs rectum, bladder, and spinal cord, no difference in dose volume parameters was found with the change in the field width.

### D. Treatment time

A 2.5 cm field width plan resulted in beam‐on treatment time of 16.19±1.3 minutes as compared to 9.1±0.7 minutes with a 5 cm beam width plan. For 2.5 cm field width plan, a modulation factor of 3.0 was used; however, only 2.4±0.05 was used by the optimizer. Similarly, for 5.0 cm beam width plan, modulation factor of 2.47±0.07 was used by the optimizer, although 3.5 was set by the planner.

## IV. DISCUSSION

The role of radiation in epithelial ovarian cancers is evolving. With newer radiation techniques like fixed‐beam IMRT, IMAT, and HT, it is possible to treat large multiple targets with simultaneous integrated boosts with optimal sparing of critical structures.^(^ ^8^ ^–^ ^10^ ^)^ To evaluate the potential of HT for WAR in ovarian cancers, we undertook this dosimetric study.

Traditionally, conventional radiotherapy techniques for WAR having AP/PA fields using 6–15 MV photon beams with partial kidney and liver shields has major limitations both in terms of target coverage and organ sparing.^(^ ^6^ ^,^ ^21^ ^)^ To improve the homogeneity and sparing of OARs, a novel method called moving strip technique has been tried with limited success.^(3)^ Fixed‐beam IMRT techniques often require two isocenters to cover the large PTV with intensity feathering.^(8)^

Generation of fixed‐beam IMRT and IMAT plans on the same patient's data set would have minimized the interinstitution uncertainties. Due to longer lengths of PTV(>40cm), lack of software support for the current TPS used for IMRT, and non‐availability of IMAT, we could not perform fixed‐beam IMRT and IMAT plans successfully. Hence, we compared our HT doses with other published modalities treating WAR, taking into consideration such issues as the total dose delivered, the percentage received by normal tissues, and the variation in volume delineation. The purpose of comparison with other modalities was to explore the dose levels that are generally acceptable and/or achievable for target as well as for OARs for WAR. An attempt was made in the present study to improve dose‐volume parameters such that maximum target coverage and OAR sparing could be achieved.

Table 2 shows the comparison of WAR with fixed‐beam IMRT, IMAT and HT techniques with respect to doses received by the PTV and OARs.

**Table 2:** Dose volume parameters of the PTV and the OARs of the present study in comparison with other published literature.

	*Duthoy et al.* ^(9)^	*Hong et al.* ^(8)^	*Garsa et al.* ^(22)^	*Rochet et al.* ^(10)^	*Present Study*
Technique	IMAT	IMRT	IMRT with gating	HT	HT
Dose to Abdomen Gy	33	30	30	30	25
Dose to Pelvis Gy	33	30	44.4	30	45
${\text{PTV‐V}}_{95\%}$	82.2	84	90	86.9	95.6
$D_{\text{mean}}\;\text{kidney}\;\%$	45	53.6	53.6	31.5	38
$D_{\text{mean}}\;\text{liver}\;\%$	74	95	100	71.9	(100) 70.4^*^
$D_{\text{mean}}\;\text{bone marrow}\;\%$	not reported	62	83	35	60

^*^ Mean dose to the liver and normal liver was 100% and 70.4%, respectively.

### A. PTV coverage

In the present study, $V_{95\%}$ was found to be more than 95% effective, much superior to other planning studies with 83.5%, 82.2% and 86.9 % in fixed‐beam IMRT, IMAT and HT, respectively.^(^ ^8^ ^–^ ^10^ ^)^ It was suggested that increase in the number of beams with fixed‐beam IMRT could improve the coverage and homogeneity.^(8)^ The coverage and homogeneity with HT is superior possibly due to inherent characteristics of multiple beamlets (spaced every seven degrees) and high modulation. $V_{95\%}$ in the present study is superior to HT results as noted in published literature, which could be attributed to the differences in the volumes and the dose volume objectives set for the optimizer.^(10)^ In the current study, optimal PTV coverage was given a higher priority following which OARs were penalised without compromising the coverage. However CI (1.077 vs 1.202) and other dose‐volume parameters (such as $V_{107\%},\;D_{\max},\;D_{\min})$ were comparable.^(10)^

### B. OARs sparing

Patients with epithelial ovarian cancers receive chemotherapy after surgery. They receive further 2nd / 3rd / 4th line chemotherapy for relapses. Consequently, preservation of hepato‐renal functions and bone marrow reserves becomes vital for patients being planned for large field abdominal radiation. Hence, our aim was to achieve optimal sparing of kidneys, liver, and bone marrow. The median dose to the kidneys in the present study was 9.6±1.2Gy, which is much lower compared to fixed‐beam IMRT and IMAT techniques (16.1Gy and 15Gy, respectively^(^ ^8^ ^,^ ^9^ ^)^) and comparable with the HT (9.45 Gy).^(10)^ Despite inclusion of antero‐lateral surfaces of both the kidneys similar to Duthoy et al.,^(9)^ optimal sparing of kidneys was achieved with HT.

The mean dose to the liver in fixed‐beam IMRT and IMAT was 90%–100% and 74% of prescribed dose, respectively.^(^ ^8^ ^,^ ^9^ ^)^ Mean liver dose was reported as 21.6 Gy (71.9%) in published HT data, as compared to 25.2 Gy (100%) in the current study.^(10)^ High liver doses in the present study could be due to the inclusion of 1–1.5 cm of liver surface in the PTV. Treatment of capsule and rim of hepatic parenchyma with margins for liver movements is crucial. Undersurface of the diaphragm, liver surfaces, and hepato‐renal fossa are the tumor sanctuary sites which are invariably untouched during surgery. In order to achieve adequate doses to these sites and at the same time minimize liver doses, a structure named ‘normal liver’ was made that consists of liver excluding PTV. The mean dose to whole liver is higher (25.2 Gy) because of adequate doses to liver other than normal liver, while the mean dose to ‘normal liver’ is only 18.3 Gy (70.4%), which is comparable to data in the published literature.^(10)^ Also, the volumes contoured are different in various studies; therefore, direct comparisons are not conclusive. However, assuming the worst case scenario of inclusion of liver surface in the PTV, the doses achieved in the present study were comparable with all the published series. Further reduction of liver doses in the present study may be possible with the use of higher modulation factor. Rochet et al.^(10)^ used a modulation factor of 4 and a field width of 2.5 cm while, in the present study, a modulation factor of 3.5 and a field width of 5.0 were used.

Mean volume of bone marrow contoured in the current study is 1345±165cc, which is larger compared to reported literature.^(8)^ This is because in the current study, bone marrow has been extensively contoured including ribs, vertebrae, pelvic bones, and upper end femurs. This differs from Hong et al.^(8)^ who included only pelvic bone and femurs. Mean dose to bone marrow was 60%, versus 35% in the Rochet study.^(10)^ Mean $V_{20\text{Gy}}$ was 84% when compared to 34.5% and 71% of prescription dose with fixed‐beam IMRT or respiratory gated fixed‐beam IMRT, respectively.^(^ ^8^ ^,^ ^22^ ^)^ The mean dose to the bone marrow was 22±1.4Gy as compared to 10.6 Gy in previously published data of HT.^(10)^ This large difference could be attributed to two reasons: (1) our contouring was extensive and is a true representation of actual doses received by the bone marrow, and (2) our dose prescription was 45 Gy to pelvis while it was 30 Gy in other study.^(10)^

### C. Comparison of MUs, treatment time and other technical parameters

It was reported that a mean of 1442 MUs was needed for a fraction dose of 150 cGy with fixed‐beam IMRT while, for IMAT, 444 MU was needed to deliver the same dose.^(^ ^8^ ^,^ ^9^ ^)^ In our study, 2.5 cm beam width plan resulted in approximately 13,000 MU with a treatment time of 16.2 minutes (compared to 7000 MU with a treatment time of 9.1 minutes with 5.0 cm beam width plan) to deliver 100 cGy to abdomen and 180 cGy to pelvic boost fields. The longer treatment time and large MU in HT may be attributed to the inherent characteristics of tomotherapy such as high modulation and the slice delivery.

It is important to emphasize here that, for technical reasons, fixed‐beam IMRT and IMAT techniques are difficult to implement in clinical practice while tomotherapy is simple to implement for large volume tumors due to its unique design.^(^ ^8^ ^,^ ^9^ ^)^ Fixed‐beam IMRT has issues of multiple isocentres, junction, and field matching. The treatment time is an issue with the fixed‐beam IMRT technique too, as most of the treatment fields were divided into multiple segments due to excessive width.^(8)^ It was also suggested that the plan quality could be improved with more than five gantry angles, but was restricted due to long treatment time, especially for patients with two isocentres. The issues in the implementation of IMAT for long treatment length are described in detail by Duthoy et al.^(9)^ New delivery techniques, such as volumetric arc therapy with variable gantry speed and dose rate, have to be investigated to evaluate whether these modalities will improve the plan quality in these patients. However, in tomotherapy it is possible to treat the entire length without any issues of multiple isocenter and junction with modest increase in treatment time. HT in clinical use is still relatively new. Further progress in hardware and software developments may result in improving the MU efficiency and treatment time without compromising the existing plan quality.

## V. CONCLUSIONS

Helical tomotherapy achieves an excellent coverage of abdominal PTV with simultaneous pelvic boost and better organ sparing. Helical tomotherapy has the advantages of being able to treat longer field lengths, and large and complex volumes. To validate further, we are currently conducting a phase II cohort study with image guidance as consolidation in epithelial ovarian cancers.
